# Effects of *Citrus aurantium* (Bitter Orange) Fruit Extracts and *p*-Synephrine on Metabolic Fluxes in the Rat Liver

**DOI:** 10.3390/molecules17055854

**Published:** 2012-05-16

**Authors:** Jéssica Sereno Peixoto, Jurandir Fernando Comar, Caroline Tessaro Moreira, Andréia Assunção Soares, Andrea Luiza de Oliveira, Adelar Bracht, Rosane Marina Peralta

**Affiliations:** Department of Biochemistry, University of Maringá, Avenida Colombo 5790, 87020900 Maringá, Brazil; Email: jessica_nutri@hotmail.com (J.S.P.); jfcomar@uem.br (J.F.C.); ca_tessaro@yahoo.com.br (C.T.M.); andasoares7@gmail.com (A.A.S.); andreabiomed@gmail.com (A.L.O.); rmperalta@uem.br (R.M.P.)

**Keywords:** adrenergic signaling, respiration, glycogenolysis, glycolysis, weight loss

## Abstract

The fruit extracts of *Citrus aurantium* (bitter orange) are traditionally used as weight-loss products and as appetite supressants. An important fruit component is *p*-synephrine, which is structurally similar to the adrenergic agents. Weight-loss and adrenergic actions are always related to metabolic changes and this work was designed to investigate a possible action of the *C. aurantium* extract on liver metabolism. The isolated perfused rat liver was used to measure catabolic and anabolic pathways, including oxygen uptake and perfusion pressure. The *C. aurantium* extract and *p*-synephrine increased glycogenolysis, glycolysis, oxygen uptake and perfusion pressure. These changes were partly sensitive to α- and β-adrenergic antagonists. *p*-Synephrine (200 μM) produced an increase in glucose output that was only 15% smaller than the increment caused by the extract containing 196 μM *p*-synephrine. At low concentrations the *C. aurantium* extract tended to increase gluconeogenesis, but at high concentrations it was inhibitory, opposite to what happened with *p*-synephrine. The action of the *C. aurantium* extract on liver metabolism is similar to the well known actions of adrenergic agents and can be partly attributed to its content in *p*-synephrine. Many of these actions are catabolic and compatible with the weight-loss effects usually attributed to *C. aurantium*.

## 1. Introduction

**Figure 1:**
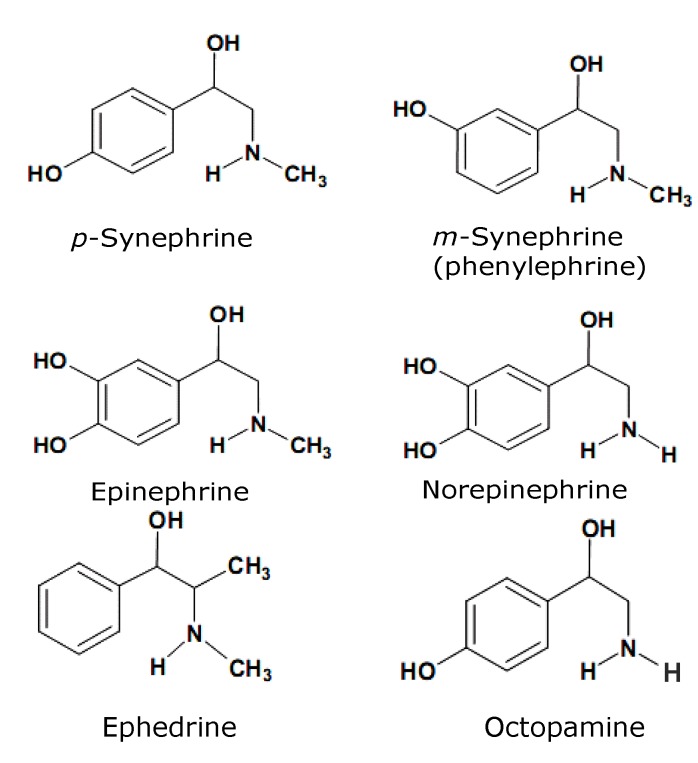
Structural formulae of the *C. aurantium* alkaloids synephrine, octopamine and related amines.

*Citrus aurantium* (Rutaceae) is popularly known as “bitter orange” and its fruit extracts are being marketed and traditionally used as herbal weight-loss products and as appetite suppressants, although in Traditional Chinese Medicine they are prescribed in concert with other support herbs [1,2,3,4]. More recently bitter orange extracts have been demonstrated to possess anxiolytic and sedative properties [5]. The alkaloids *p*-synephrine and to a lesser extent octopamine (Figure 1) are believed to be the most active components of *C. aurantium*. However, the peels of the fruits also contain terpenes, furocumarines and flavonoids, including hesperidin, neohesperidin, naringin and tangaretin [6,7]. The *p*-synephrine content of the dried fruit extracts has been reported to be between 3% to 6% [8,9,10,11]. The alkaloids of *C. aurantium* are structurally related to the adrenergic amines ephedrine (*Ephedra sinica*), epinephrine, norepinephrine and *m*-synephrine (Figure 1). The latter is a vasoactive isomer of *p*-synephrine whose presence in *C. aurantium* has been suggested, but not confirmed [12,13]. Like the other adrenergic amines, *p*-synephrine is believed to act via α- and β-adrenoreceptors [2,14,15]. Lower binding affinities of these receptors for *p*-synephrine, however, have been reported when compared to norepinephrine and *m*-synephrine, for example. There are well fundamented suggestions, however, that binding of *p*-synephrine to β_3_ adrenoreceptors, which are associated to thermogenesis and lipolysis, could be a significant factor of the physiological action of the compound [15]. *C. aurantium* extracts marketed for weight-loss are prepared from dried fruit peels and may be associated with other compounds, mainly caffeine [16]. Despite its inclusion in over-the-counter weight-loss products, little evidence supports the use of *C. aurantium* alone to treat overweight and obesity [11]. It has been shown that extracts increase lipolysis, thermogenesis and weight loss in both animal models and humans, but always tested in combination with other compounds, especially caffeine, which is also known to enhance the thermogenic properties of herbal weight-loss preparations [2,11]. As the *C. aurantium* extracts, *p*-synephrine effectively promoted weight loss or thermogenesis in human models only in combination with other stimulants [17,18,19], although in pure form it was effective in mice and rats [4,8,20]. 

Weight loss is always associated to metabolic effects. The liver is the metabolic organ par excellence and one can expect that *p*-synephrine, as an adrenergic agonist, should affect liver metabolism. It is known, for example, that epinephrine, norepinephrine and other adrenergic agonists stimulate glycogenolysis and gluconeogenesis in the liver via α- and β-adrenoreceptors [21,22,23,24,25,26,27,28]. Up to now there are no studies about the action of *C. aurantium* and *p*-synephrine on liver metabolism and the present work reports our first attempts at addressing this matter. More specifically, we have used the isolated perfused rat liver to investigate the actions of *C. aurantium* extracts and *p*-synephrine on glycogenolysis, glycolysis and gluconeogenesis. Additionally we have also measured portal perfusion pressure and oxygen uptake, the latter being a parameter that in the liver is largely dependent on the oxidation of fatty acids. An important goal of our study was to find out if the effects of a commercial *C. aurantium* extract*,* the most usual formulation that is consumed for therapeutic purposes, can be explained basically for its *p*-synephrine contents.

## 2. Results and Discussion

### 2.1. Effects of *C. aurantium* on Glycogen Catabolism, Glycolysis, Oxygen Uptake

**Figure 2:**
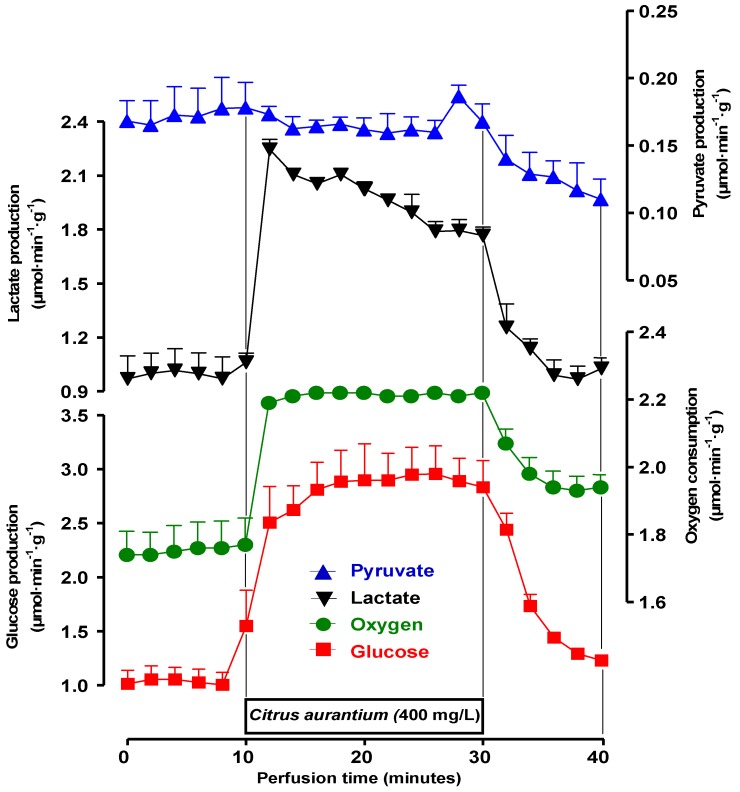
Time courses of the changes caused by the *C. aurantium* extract (400 mg/L) on glycogen catabolism and endogenous fatty acids-driven oxygen uptake. Livers from fed rats were perfused as described in the Experimental Section. *C. aurantium* (400 mg/L) was infused during 20 min, as indicated by the horizontal bar. The efﬂuent perfusate was sampled in 2-min intervals and analyzed for glucose, lactate, and pyruvate. Oxygen consumption was followed polarographically. Each datum point represents the means of three liver perfusion experiments. Bars are standard errors of the mean.

The first experiments were planned in order to test the possible effects of *C. aurantium* on glycogen catabolism and glycolysis. When perfused with substrate-free medium the livers from fed rats survive at the expense of the oxidation of endogenous fatty acids (major route) and glycolysis from endogenous glycogen (minor route). Under these conditions the livers release glucose, lactate and pyruvate as a result of glycogen catabolism [29]. Figure 2 illustrates the responses of perfused livers to the infusion of a *C. aurantium* extract at the concentration of 400 mg/L. It also illustrates a typical experimental protocol, which was used for all other extract concentrations. After a pre-perfusion period of 10 min, the *C. aurantium* extract was infused during 20 min. This was followed by additional 10 min of extract-free perfusion. Four parameters were measured: glucose release, lactate and pyruvate production and oxygen consumption. As noted in Figure 2, all parameters were stable before the initiation of *C. aurantium* infusion. After the onset of the infusion, however, oxygen uptake increased and remained so during the entire infusion period. Glucose release and lactate production were also increased with peak values 200 and 100% above the basal values, respectively. Pyruvate production was not changed. After removing the extract from the perfusion fluid, the stimulations of glucose release and lactate production returned to their basal levels. The recovery of oxygen consumption, however, was incomplete. 

**Figure 3:**
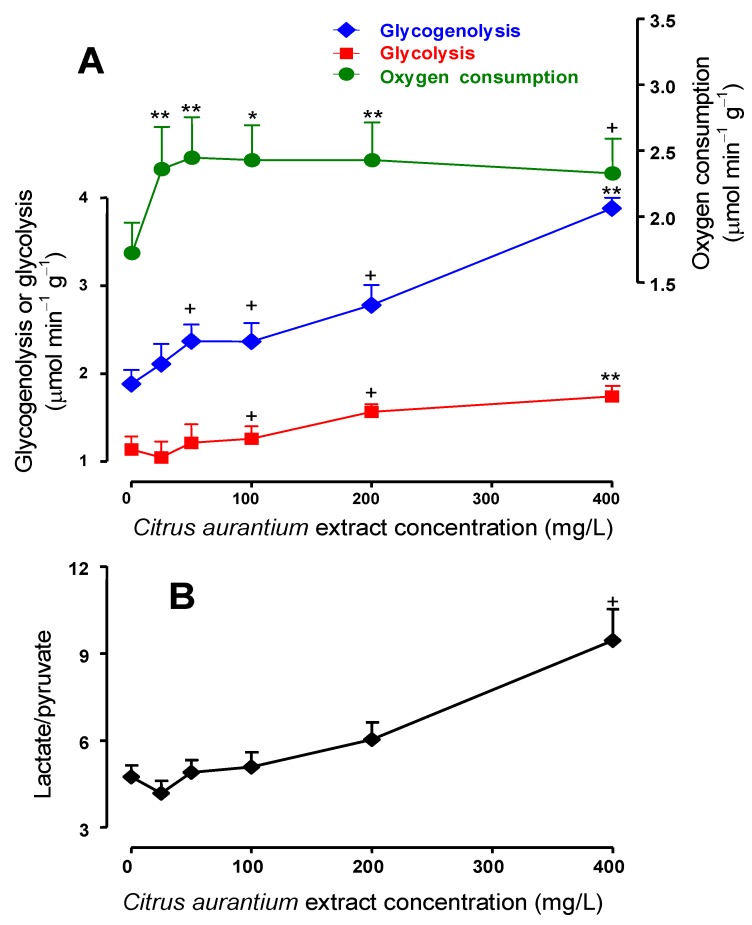
Concentration dependences of the effects of the *C. aurantium* extract on glycogen catabolism and endogenous fatty acids-driven oxygen uptake. Data were obtained from experiments of the kind illustrated by Figure 2. Glycogenolysis and glycolysis are expressed as glucosyl units and were calculated as glucose production +½(lactate production + pyruvate production) and lactate production + pyruvate production, respectively. Each datum point represents the mean of three liver perfusion experiments. Bars are standard errors of the mean. Asterisks and crosses indicate statistical signiﬁcance in comparison with the control condition as revealed by variance analysis with *post hoc* Student-Newman-Keuls testing (^+^ *p*< 0.05, ** *p* < 0.01).

Experiments like those illustrated in the Figure 2 were repeated with 25, 50, and 100 mg/L of *C. aurantium* extract in order to investigate the concentration dependence of the effects. The mean results are shown in Figure 3A and represent the final values of each parameter at the end of the drug infusion period (25–30 min perfusion time in Figure 2). Oxygen consumption, glycogenolysis (glucose plus ½[pyruvate + lactate]) and glycolysis (pyruvate plus lactate) were represented against the *C. aurantium* extract concentration. As revealed by Figure 3A, the *C. aurantium* extract increased almost linearly both glycogenolysis and glycolysis in the range up to 400 mg/L (196 μM synephrine). Oxygen uptake, however, was already maximal at the concentration of 25 mg/L (12.3 μM synephrine) with no significant modifications in the range up to 400 mg/L.Figure 3B shows the changes caused by the *C. aurantium* extract on the lactate to pyruvate ratio, which is an indicator of the cytosolic NADH/NAD^+^ ratio [30]. It is apparent that the extract produced a considerable increase in the lactate to pyruvate ratio (200% at the concentration of 400 mg/L). It is noteworthy to mention that all these effects of the *C. aurantium* extract, stimulations of glycogenolysis, glycolysis and oxygen uptake, were also reported for the adrenergic agonists epinephrine [23,24], norepinephrine [25,26] and phenylephrine [26]. These effects are different from those of glucagon which, while stimulating glycogenolysis and oxygen uptake, strongly inhibits glycolysis [23].

**Figure 4:**
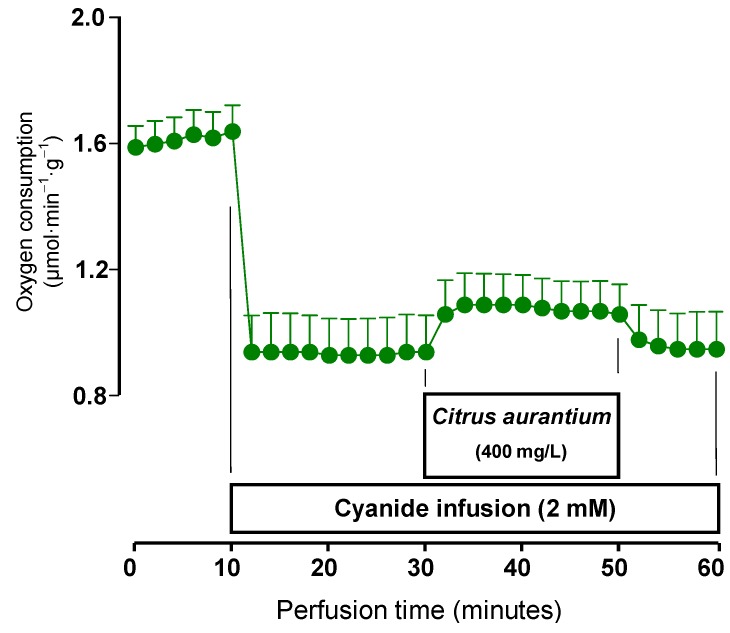
Effects of the *C. aurantium* extract on oxygen uptake in the presence of cyanide. Livers from fed rats were perfused as described in the Experimental Section. The infusion of cyanide was started after stabilization of oxygen uptake. The times at which infusion of cyanide and *C. aurantium* extracts (400 mg/L) were started are indicated. Oxygen consumption was followed polarographically. Each datum point represents the means of three liver perfusion experiments. Bars are standard errors of the mean.

To verify if oxygen uptake stimulation comes from the mitochondria, the microsomal electron transport chain or from both, experiments were done with cyanide, which at the concentration of 2 mM completely blocks the mitochondrial respiratory chain [29]. The results are shown in Figure 4. As expected cyanide greatly inhibited oxygen uptake. The infusion of the *C. aurantium* extract in the presence of cyanide showed only a discrete increase in oxygen uptake. This is strongly indicative for a predominantly mitochondrial origin of the *C. aurantium* effects on oxygen consumption.

Stimulation of oxygen uptake in the liver from fed rats perfused with substrate-free medium represents mainly an increased oxidation of endogenous fatty acids [29]. The source includes already existent free fatty acids as well as fatty acids arising from lipolysis [31]. The latter is stimulated by adrenergic agents, as indeed demonstrated for *p*-synephrine in isolated lipocytes [32]. This is a typically catabolic process, but it is not necessarily thermogenic in nature. In fact, it has been shown that stimulation of oxygen uptake by the adrenergic agonist epinephrine is accompanied by a significant increase in the mitochondrial ATP content without significant changes in the cytosolic content [23]. This overall increase in the cellular ATP content is, in principle, not indicative of a merely thermogenic activity of adrenergic agents in the liver.

### 2.2. Effects of *C. aurantium* on Gluconeogenesis and Oxygen Consumption Due to Lactate Infusion in Livers from Fasted Rats

**Figure 5:**
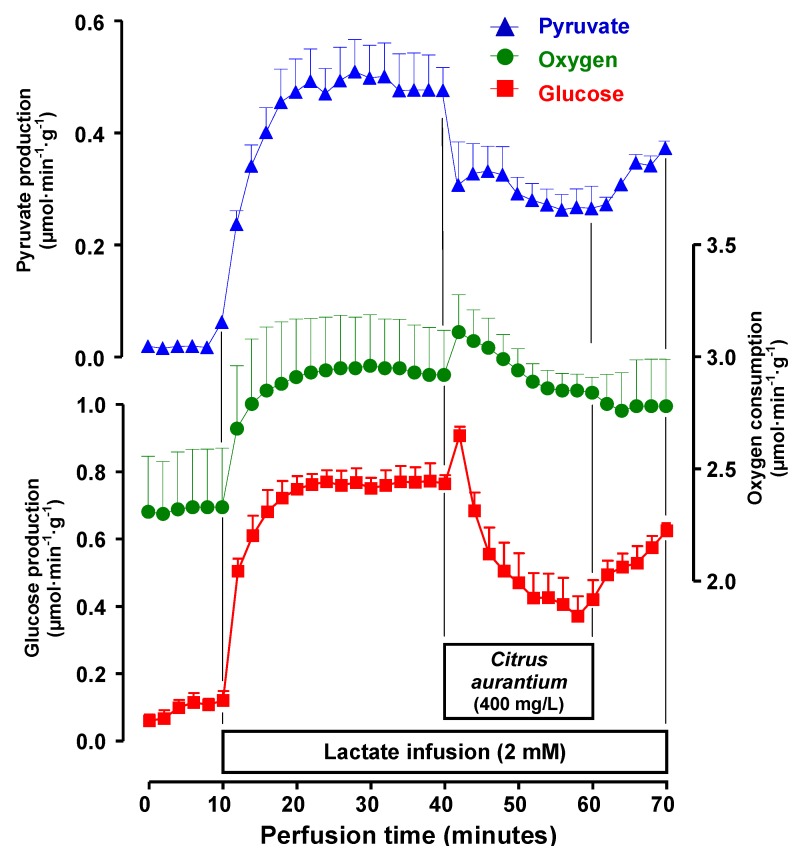
Time courses of the effects of the *C. aurantium* extract on gluconeogenesis from lactate and related parameters in perfused livers from fasted rats. Livers from fasted rats were perfused as described in the Experimental Section. Lactate (2 mM) and *Citrus aurantium* were infused as indicated by the horizontal bars. Each datum point represents the means of three liver perfusion experiments. Bars are standard errors of the mean.

The experiments shown in Figure 5 were planned to investigate a possible action of *C. aurantium* on gluconeogenesis from lactate. In order to minimize interference by glycogen catabolism, livers from 18 h fasted rats were used. Figure 5 illustrates the responses of the perfused livers to the *C. aurantium* extract infusion at the concentration of 400 mg/L. After a pre-perfusion period of 10 min with substrate-free medium, 2 mM lactate was infused during 30 min, followed by additional 20 min of *C. aurantium* extract plus lactate infusion. The infusion of 2 mM lactate produced a rapid and sustained increase in glucose and pyruvate production and also in oxygen uptake. The infusion of 400 mg/L *C. aurantium* produced non-significant oscillations in oxygen uptake and inhibitions of pyruvate and glucose production. The latter was more strongly inhibited than the former. 

**Figure 6:**
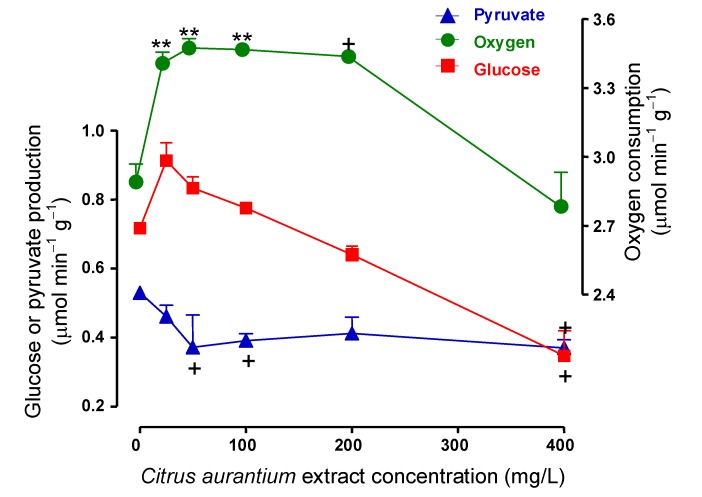
Effects of the *C. aurantium* extract on gluconeogenesis and related parameters: concentration dependences. The experimental protocol was the same illustrated by Figure 5. Each datum point is the mean of three experiments. Asterisks and crosses indicate statistical signiﬁcance in comparison with the control condition as revealed by variance analysis with *post hoc* Student-Newman-Keuls testing (^+^ *p* < 0.05, ** *p* < 0.01).

The effects on lactate metabolism caused by the *C. aurantium* extract at the concentration of 400 mg/L might not be the same at lower concentrations. For this reason the experiments illustrated by Figure 5 were repeated with extract concentrations in the range between 25 and 200 mg/L. The results are summarized in Figure 6 which shows the final values of the variables measured at the end of the *C. aurantium* extract infusion period (50–60 min perfusion time) as a function of the concentration. Figure 6 shows that the *C. aurantium* extract clearly increased oxygen uptake in the concentration range between 25 and 200 mg/L. Inhibition occurred only at the concentration of 400 mg/L. This was also the only concentration that produced a clear inhibition of glucose production. At the lowest extract concentrations (especially 25 mg/L) a stimulatory tendency was apparent, but statistical significance was lacking. Pyruvate production, finally, was clearly inhibited at low extract concentrations, maximal inhibition occurring at the concentration of 50 mg/L.

### 2.3. Effects of *p*-Synephrine on Glycogen Catabolism, Oxygen Uptake and Gluconeogenesis

**Figure 7:**
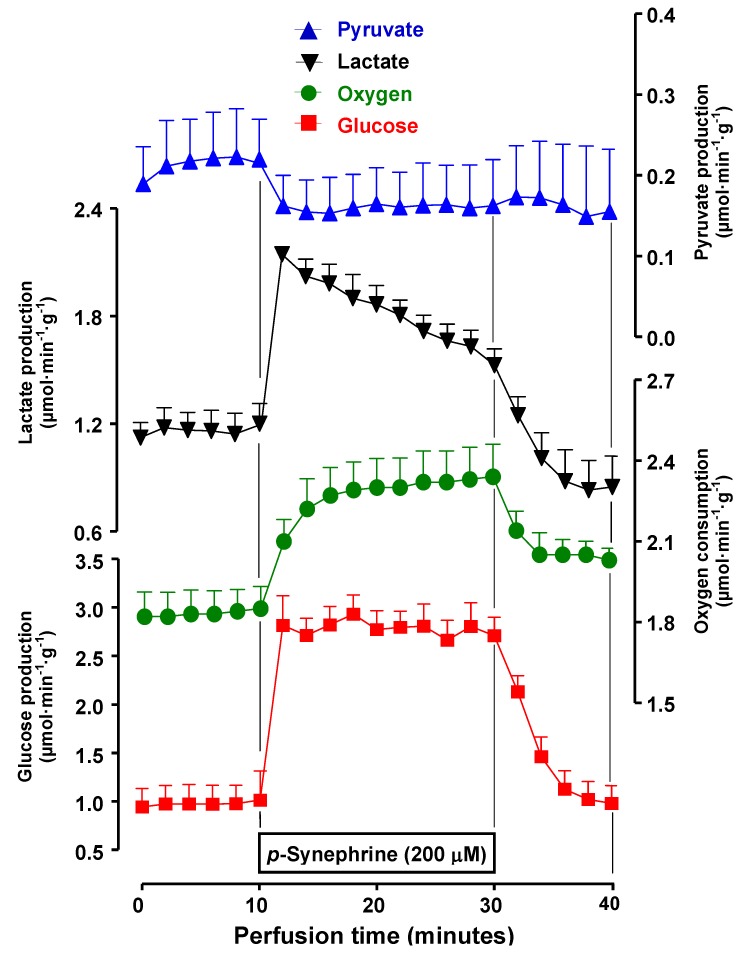
Time courses of the effects of 200 µM *p*-synephrine on glycogen catabolism and fatty acid-driven oxygen uptake. Livers from fed rats were perfused as described in the Experimental Section. *p*-Synephrine (200 µM) was infused during 20 min, as indicated. The efﬂuent perfusate was sampled in 2-min intervals and analyzed for glucose, lactate and pyruvate. Oxygen consumption was followed polarographically. Each datum point represents the means of three liver perfusion experiments. Bars are standard errors of the mean.

Since *p*-synephrine is the main active component of *C. aurantium*, experiments were designed to investigate the effect of *p*-synephrine. The experimental protocols of these experiments were similar to those used in the experiments with the *C. aurantium* extracts. The *p*-synephrine concentration was 200 μM, which is practically the same molar concentration of this component that can be expected when the extract is infused at a concentration of 400 mg/L (196 μM). Figure 7 shows the results of the experiments in which glycogen catabolism and oxygen uptake in livers from fed rats were measured. Comparison of Figure 2 and Figure 7 reveals immediately that the effects of 400 mg/L extract and of 200 μM *p*-synephrine were very similar in qualitative terms. Quantitatively, however, some differences were observed. *p*-Synephrine (200 μM) produced an increase in glucose output of 1.73 μmol·min^−1^·g^−1^, an effect that was only 15% smaller than the increment caused by the extract (400 mg/L) containing 196 μM *p*-synephrine, which was equal to 2 μmol·min^−1^·g^−1^. For oxygen consumption stimulation, however, the difference was much more pronounced, 0.28 μmol·min^−1^·g^−1^ for *p*-synephrine and 0.45 μmol·min^−1^·g^−1^ for the extract (60% difference). It seems, thus, that *p*-synephrine is not the only agent in the *C. aurantium* extract capable of changing metabolic parameters in the liver.

**Figure 8:**
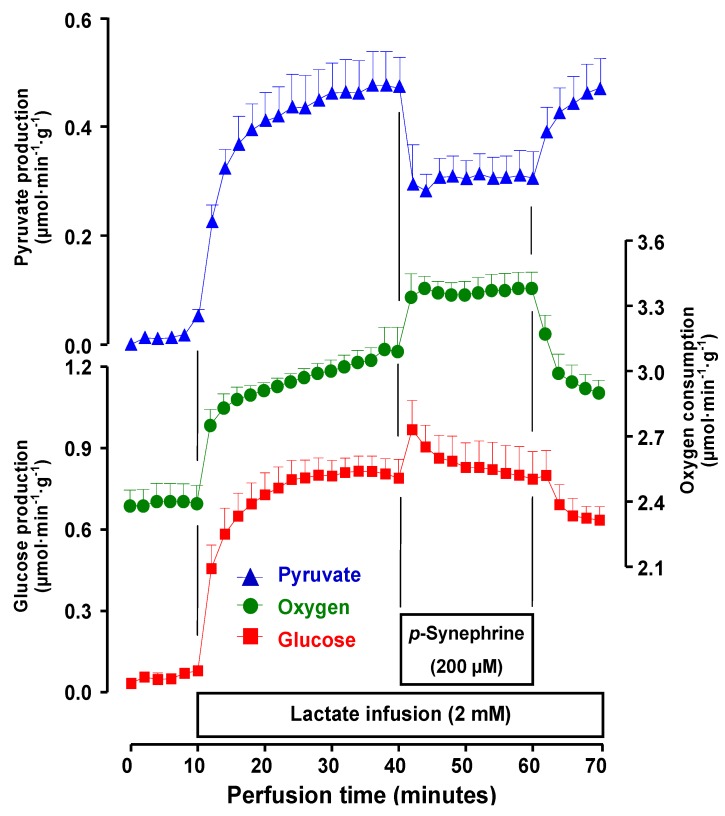
Effects of *p*-synephrine on lactate gluconeogenesis and related parameters in perfused livers from fasted rats. Livers from fasted rats were perfused as described in the Experimental Section. Lactate (2 mM) and *p*-synephrine were infused as indicated by the horizontal bars.

The effects of 200 μM *p*-synephrine on lactate gluconeogenesis were also investigated. The results are shown in Figure 8 . In this case comparison of Figure 5 and Figure 8 reveals different actions of the extract containing 196 μM *p*-synephrine and 200 μM pure *p*-synephrine, except for pyruvate production which was inhibited. *p*-Synephrine did not inhibit gluconeogenesis, actually a clear stimulatory tendency which persisted during the whole infusion period was apparent. Oxygen uptake was also stimulated during the whole perfusion period of *p*-synephrine. These results strongly corroborate the hypothesis raised above that not all effects of the extract are caused by *p*-synephrine. However, it should be recalled that the lactate stimulated respiration was not inhibited by the extract at low concentrations (Figure 6).

Actually stimulation was found. Furthermore, inhibition of gluconeogenesis, which is not an adrenergic effect, occurred only at the highest extract concentration (400 mg/L). This suggests that the additional and non-adrenergic effects of the extract only manifest themselves at high concentrations. They could well be non-significant under in vivo conditions.

### 2.4. Influence of Adrenergic Antagonists on the Actions of the *C. aurantium* Extract

In order to obtain a first insight about the involvement of adrenergic signaling in the actions of the *C. aurantium* extract experiments were done in which the influence of four adrenergic antagonists was quantified. The antagonists were prazosin (α_1_ antagonist; [22]), yohimbine (α_1_ and α_2_ antagonist; [22]) propranolol (β_1_ and β_2_ antagonist; [21]) and SR59230A (a β_3_ antagonist; [33]). The results are shown in Table 1. In this series of experiments the portal perfusion pressure was also measured taking into account the fact that adrenergic agonists are generally vasoactive [22,27]. The experimental protocol was similar to that one illustrated by Figure 2, with a pre-perfusion period for evaluating the basal rates and portal perfusion pressure and a subsequent infusion of the *C. aurantium* extract (400 mg/mL) during 20 min. For testing the action of the adrenergic antagonists these substances were infused alone during 10 min prior to initiating the *C. aurantium* extract infusion (400 mg/mL). The infusion of the antagonists was continued during the *C. aurantium* extract infusion. The parameters that were monitored were evaluated in terms of the differences between the values found in the simultaneous presence of the *C. aurantium* extract and the antagonist and the values found before the extract infusion. None of the antagonists had any effect on the metabolic parameters or perfusion pressure in the absence of the *C. aurantium* extract (not shown). However, most of them had some influence on the effects of the *C. aurantium* extract. The notable exception was SR59230A, which had no significant effects on both metabolic parameters and hemodynamics. The basal portal perfusion pressure was equal to 3.63 ± 0.13 mm Hg. The modification caused by the *C. aurantium* extract shown in Table 1 represents, thus, a 2.6-fold increase. 

**Table 1:** Effects of various antagonists on the effects of the *Citrus aurantium* extract on glycogen catabolism, oxygen uptake and perfusion pressure. Livers from fed rats were perfused with Krebs/Henseleit-bicarbonate. For the control experiments, the experimental protocol illustrated by Figure 2 was used. In the experiments in which the antagonists were infused, the *Citrus aurantium* extract infusion was preceded by the infusion of one of the three antagonists (10 min). The concentrations were: *Citrus aurantium* extract, 400 mg/mL;prazosin, 10 μM; yohimbine, 100 μM; propranolol, 50 μM; and SR59230A, 10 μM. Glucose and lactate production and oxygen uptake were measured as described in the Experimental Section. The portal perfusion pressure was monitored by means of a pressure transducer. Values represent the mean increments (± mean standard errors; n = 3–4) produced by the *Citrus aurantium* extract under the various conditions. Asterisks indicate statistical signficance at the 5% level when compared to the control condition (*Citrus aurantium* extract alone) as revealed by variance analysis with post-hoc testing (Student-Newman-Keuls).

Perfusate conditions	Δ Portal pressure (mm Hg)	Δ Oxygen uptake (μmol min^−1^ g^−1^)	Δ Glucose release (μmol min^−1^ g^−1^)	Δ Lactate production (μmol min^−1^ g^−1^)
*Citrus aurantium* extract alone (control)	5.81 ± 1.00	0.54 ± 0.08	1.86 ± 0.12	0.80 ± 0.08
*Citrus aurantium* extract + prazosin	0.92 ± 0.01 *	0.44 ± 0.09	0.32 ± 0.03 *	0.26 ± 0.09 *
*Citrus aurantium* extract + yohimbine	0.10 ± 0.04 *	0.22 ± 0.03 *	0.04 ± 0.01 *	0.38 ± 0.04 *
*Citrus aurantium* extract + propranolol	2.60 ± 0.75 *	0.31 ± 0.01	0.34 ± 0.03 *	0.55 ± 0.14
*Citrus aurantium* extract + SR59230A	5.99 ± 0.85	0.53 ± 0.03	1.88 ± 0.14	0.60 ± 0.17

Yohimbine almost abolished this increase and prazosin had also a strong inhibitory effect. The action of propranolol was much less pronounced. Stimulation of glucose release (glycogenolysis) was also almost abolished by yohimbine, and strongly inhibited by both prazosin and propranolol. Oxygen uptake stimulation was much less sensitive to the antagonists and the action of prazosin and propranolol even lacked statistical significance. Lactate production (glycolysis) was more strongly affected by prazosin. The actions of the adrenergic antagonists in Table 1 present a relatively complex picture but they certainly indicate that both α- and β-adrenergic receptors are involved in the actions of the *C. aurantium* extract. It can even be said that the participation of β-adrenergic receptors is less important in the case of the portal pressure increase and the stimulation of lactate production. The exact contribution of α- or β-receptors, however, cannot be inferred from the present data. This is a question that requires exhaustive experimental work in which the actions of individual compounds found in *C. aurantium* (e.g., *p*-synephrine and octopamine) must be examined for their sensitivity to specific antagonists of the various classes of adrenergic receptors. Cross-talking between various signaling pathways is also a highly probable event which deserves attention. The lack of effects of SR59230A indicates, in principle at least, that the hepatic effects observed in the present work are not elicited by binding of *p*-synephrine to β_3_-adrenergic receptors although there is evidence that these receptors are present in the liver [34,35]. It should be stressed, however, that it seems well documented that *p*-synephrine acts via these receptors in other tissues [15]. 

## 3. Experimental

### 3.1. Materials and Animals

The liver perfusion apparatus was built in the workshops of the University of Maringá. *p*-Synephrine, enzymes and coenzymes used in the enzymatic assays were purchased from Sigma-Aldrich Co (St. Louis, MO, USA). Commercial *C. aurantium* dried extracts were purchased from the company Pharma Nostra (Rio de Janeiro, Brazil; lot ZS071226). The extracts were manufactured by the Shanxi Company (China, lot 08041136D-2008). The manufacturer indicates that the concentration of *p*-synephrine in the extracts is higher than 6% and chemical analysis by Pharma Nostra indicated 8.2% synephrine. All other chemicals were from the best available grade (98–99.8% purity) and were purchased from Sigma-Aldrich, Merck (Darmstadt, Germany) and Reagen ( Rio de Janeiro, Brazil).

Male Wistar rats weighing 200–280 g were used in all experiments. Animals were fed *ad libitum* with a standard laboratory diet (Nuvilab^®^, Colombo, Brazil) and maintained on a regulated light-dark cycle. In accordance with the requirements of the experimental protocols, fed rats as well as 18 h fasted rats were used. For the surgical procedure, the rats were anesthetized by intraperitoneal injection of sodium pentobarbital (50 mg/Kg). The criterion of anesthesia was the lack of body or limb movement in response to a standardized tail clamping stimulus. All experiments were done in accordance with the world-wide accepted ethical guidelines for animal experimentation.

### 3.2. Liver Perfusion

Hemoglobin-free, non-recirculating perfusion was performed [29,36]. After cannulation of the portal and cava veins the liver was positioned in a plexiglass chamber. The constant perfusate flow was provided by a peristaltic pump (Minipuls 3, Gilson, Villier Le Bel, France) and adjusted between 30 and 33 mL per min, depending on the liver weight. The perfusion fluid was Krebs/Henseleit-bicarbonate buffer (pH 7.40) containing 25 mg% bovine-serum albumin, saturated with a mixture of oxygen and carbon dioxide (95:5) by means of a membrane oxygenator with simultaneous temperature adjustment (37 °C). The composition of the Krebs/Henseleit-bicarbonate buffer is the following: 115 mM NaCl, 25 mM NaHCO_3_, 5.8 mM KCl, 1.2 mM Na_2_SO_4_, 1.18 mM MgCl_2_, 1.2 mM NaH_2_PO_4_ and 2.5 mM CaCl_2_. The perfusion fluid enters the liver via the portal vein cannula and leaves the organ through the cava vein cannula. Samples of the effluent perfusion fluid were collected and analyzed for their metabolite contents. *C. aurantium* and *p*-synephrine were added to the perfusion fluid at the desired concentrations (up to 200 mg/L).

### 3.3. Analytics

In the effluent perfusion fluid the following compounds were assayed by means of standard enzymatic procedures: glucose, lactate and pyruvate [37]. The oxygen concentration in the outflowing perfusate was monitorated continuously, employing a Teflon-shielded platinum electrode adequately positioned in a plexiglass chamber at the exit of the perfusate [29,36]. Metabolic rates were calculated from input-output differences and the total flow rates and were referred to the wet weight of the liver.

The portal perfusion pressure was monitored by means of a pressure transducer (Hugo Sachs Elektronic-Harvard Apparatus GmbH, March-Hugstetten, Germany). The sensor was positioned near the entry of the portal vein, and the transducer was connected to a recorder [38]. The pressure changes were computed from the recorder tracings and expressed as millimeters of mercury (mm Hg).

### 3.4. Statistical Analysis

The error parameters presented in the graphs are standard errors of the means. Statistical analysis was performed with the GraphPad Prism Software^®^ (version 5.0; Graph Pad Software, San Diego, CA, USA). The Student’s *t* test was applied and the 5% level (*p* < 0.05) was adopted as a criterion of significance.

## 4. Conclusions

The results of the present work reveal that the metabolic action of the *C. aurantium* extract on liver metabolism and portal perfusion pressure is similar to the actions of other adrenergic agents. The same applies to *p*-synephrine, which is generally considered to be the main active component of *C. aurantium*. The sensitivity to adrenergic agonists is strong evidence that several effects are indeed mediated by adrenergic signaling pathways. Since many of the actions of *C. aurantium* are mainly catabolic in nature, e.g., increased glycolysis and oxygen uptake dependent on fatty acid oxidation, it can be concluded that they are, in principle at least, compatible with the weight-loss effects usually attributed to *C. aurantium*. 
